# All night analysis of time interval between snores in subjects with sleep apnea hypopnea syndrome

**DOI:** 10.1007/s11517-012-0885-9

**Published:** 2012-03-10

**Authors:** J. Mesquita, J. Solà-Soler, J. A. Fiz, J. Morera, R. Jané

**Affiliations:** 1 Department ESAII, Universitat Politècnica de Catalunya (UPC), Barcelona, Spain; 2 Institut de Bioenginyeria de Catalunya (IBEC), Baldiri Reixac, 4, Torre I, 9 floor, 08028 Barcelona, Spain; 3 CIBER de Bioingeniería, Biomateriales y Nanomedicina (CIBER-BBN), Barcelona, Spain; 4 Servei de Pneumologia, Hospital Universitari Germans Trias i Pujol, CIBERES, Badalona, Spain

**Keywords:** Sleep apnea, Snore sounds, Snore time interval

## Abstract

Sleep apnea–hypopnea syndrome (SAHS) is a serious sleep disorder, and snoring is one of its earliest and most consistent symptoms. We propose a new methodology for identifying two distinct types of snores: the so-called non-regular and regular snores. Respiratory sound signals from 34 subjects with different ranges of Apnea-Hypopnea Index (AHI = 3.7–109.9 h^−1^) were acquired. A total number of 74,439 snores were examined. The time interval between regular snores in short segments of the all night recordings was analyzed. Severe SAHS subjects show a shorter time interval between regular snores (*p* = 0.0036, AHI cp: 30 h^−1^) and less dispersion on the time interval features during all sleep. Conversely, lower intra-segment variability (*p* = 0.006, AHI cp: 30 h^−1^) is seen for less severe SAHS subjects. Features derived from the analysis of time interval between regular snores achieved classification accuracies of 88.2 % (with 90 % sensitivity, 75 % specificity) and 94.1 % (with 94.4 % sensitivity, 93.8 % specificity) for AHI cut-points of severity of 5 and 30 h^−1^, respectively. The features proved to be reliable predictors of the subjects’ SAHS severity. Our proposed method, the analysis of time interval between snores, provides promising results and puts forward a valuable aid for the early screening of subjects suspected of having SAHS.

## Introduction

Sleep apnea–hypopnea syndrome (SAHS) is a serious sleep disorder with high community prevalence that may cause deterioration in quality of life, traffic accidents, arterial hypertension and cardiovascular and cerebrovascular diseases [22]. Furthermore, it has been demonstrated that undiagnosed patients double the expenditure of health care resources compared with diagnosed and treated patients. The gold standard for diagnosing SAHS is an overnight polysomnographic study performed at the hospital, a laborious, expensive and time-consuming procedure in which multiple biosignals are recorded [6]. Recently, several authors have suggested simplified methods to aid the screening of SAHS based on a reduced number of signals [6, 10]—or even a single one—such as ECG [1, 19], pulse oximetry [37], breath sounds [20, 37], snore sounds [9, 30] or nasal airway pressure [14].

Snoring is known to be an important clinical hallmark of SAHS [3, 28]. As such, it may be a useful and an easily accessible signal to screen this disease. Acoustic analysis of snoring reveals information relating to the site and degree of obstruction of the upper airway. For this reason, research studies on automatic detection and classification of snore sounds have received considerable attention recently [2, 9, 16, 17, 30, 34, 37]. Several acoustic markers have proven to be able to discriminate between simple snorers and SAHS patients. These markers include, but are not limited to, pitch [18]; formant frequencies [26]; peak frequencies [8, 25]; soft phonation index and noise-to-harmonics ratio [12], and even psychoacoustic metrics in terms of loudness, sharpness, roughness and annoyance [7, 13].

As an alternative to the aforementioned acoustic analysis of snoring episodes Cavusoglu et al. [5] proposed the study of snore episode separations (SES) between successive snoring episodes in the same snoring state. Since they did not succeed on automatically identifying the snoring states, they suggested overcoming this issue by considering only separations less than 10 s. Even though their results shown the strong potential of SES on distinguishing simple snorers from SAHS patients, no classification results were reported for their database of subjects.

It is important to note that snoring does not have a fixed and constant occurrence, since it is subject to many influences such as body position, sleep stages, route of breathing (oral, nasal, or both) and the degree and site of upper airway narrowing [28]. Not all snoring episodes have the same characteristics and trigger mechanisms during sleep, akin to what happens with the breathing pattern, which changes and shows irregularities during the lighter sleep stages [4]. In this way, it is crucial to make a distinction between two different types of snores: the ones that are successive and produced in consecutive breathing cycles —*regular snores*— and the ones that are separated by non-snoring breathing cycles and/or apneas—*non*-*regular snores*.

In this work, we will show that relevant information on the severity of SAHS can be estimated by the simple analysis of the time interval between regular snores, without the need to resort to any complementary and likely more complex, acoustic analysis of snores.

## Methods

### Signal acquisition

Snoring sound signals were acquired during full-night polysomnography at the sleep disorders laboratory of the *Hospital Universitari Germans Trias i Pujol* in Badalona, Spain. The snoring sound signal was recorded with an encapsulated unidirectional electric condenser microphone, placed over the trachea at the level of the cricoid cartilage, and fastened using an elastic band. A pioneer single-channel device (Snoryzer Uno; Sibel SA, Barcelona, Spain) was used to record the respiratory sounds during sleep. The sound signal was amplified, filtered between 70 and 2,000 Hz using a second order Butterworth analog band-pass filter and digitized at a sampling frequency of 5,000 Hz with a 12-bit analog to digital converter [9]. Snoring episodes and their time boundaries were identified by a previously trained and validated automatic detector and analyzer developed by our research group (DLL Snore Analyzer v9.52) [16, 17].

### Database

The database of respiratory sound signals consisted of 34 subjects (8 females and 26 males) with age range of 37–72 years and apnea–hypopnea index (AHI) range of 3.7–109.9 h^−1^. All subjects were free of any upper airway infection and other diseases throughout the study, and none had undergone treatment for snoring or were taking any medication at the time of data collection. The study was approved by the research ethics committee of the *Hospital Universitari Germans Trias i Pujol* and informed consent was obtained from all patients. The characteristics of the database, divided into two groups with opposite values of severity (above and under AHI 30 h^−1^), are described in Table 1.

**Table 1:** Characteristics of the database

	AHI	NSn	Age	BMI	Nr Subj
G AHI < 30					
m	11.8	1,752	50	26.32	16 (7F; 9 M)
s	8.3	877	12	2.8	
G AHI ≥ 30					
m	60.5	2,580	52	30.43	18 (1F; 17 M)
s	22.8	828	8	3.9	

*G* group of subjects, *AHI* apnea–hypoapnea index (h^−1^), *NSn* number of snores, *BMI* body mass index (kg/m^2^), *F* female, *M* male, *m* mean value, *s* standard deviation

### Adaptive threshold for regular snore identification

**Fig. 1:**
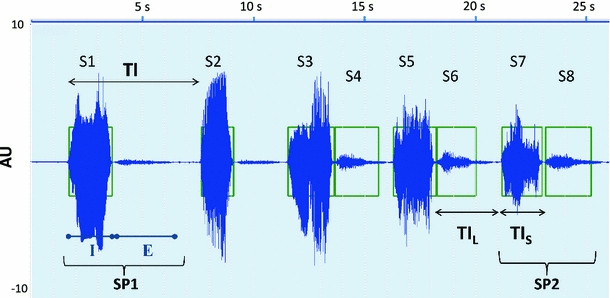
Example of a 26 s excerpt of a snoring sound signal from a subject in our database. The *boxes* indicate snore episodes. I and E stand for inhalation and exhalation, respectively. SP1 is the single snoring pattern and SP2 is the double snoring pattern

Let TI be the time interval between successive snores, calculated as the time interval between the onset of a snore and the onset of its previous one (Fig. 1):

1 $$ {\text{TI}}(i) = S_{\text{onset}} (i) - S_{\text{onset}} (i - 1)\quad i = 1, \ldots ,{\text{NSn}} $$

where *S*_onset_(*i*) is the onset of the detected *i*th snore *S*(*i*) and NSn is the total number of detected snores*.*

Snoring is mostly seen in the inspiratory phase but it can also be present in the exhalation phase [29]. We are interested in studying the snoring episodes that are produced in consecutive breathing cycles. These regular snores enclose two kinds of snoring pattern:

SP1: single pattern, when the subject snores once per breathing cycle, while inhaling or while exhaling;
SP2: double pattern, when the subject snores both in inhalation and exhalation of the same breathing cycle.

Figure 1 shows five breathing cycles. SP1 is present on the two first breathing cycles and SP2 is present on the last three. S7 and S8 correspond, respectively, to inhaling and exhaling snores of the same breathing cycle. Hence, they constitute the snoring pattern SP2, where S7 has a longer time interval (TI_L_) and S8 has a shorter time interval (TI_S_).

We used a previously proposed adaptive threshold TH_adaptive_ [23, 24] to identify snoring episodes that are truly consecutive, i.e., two snores that are neither separated by an apnea event nor separated by non-snoring breathing cycles. This threshold is adaptively estimated from the whole night sequence of time intervals between successive snores. As a result, it is characteristic of the particular snoring pattern of each subject. To compute the threshold only one initial condition is set: the threshold is initialized with value 10 (θ = 10 s) because the first few snores produced during sleep are not descriptive of the subjects’ snoring pattern during the night and could introduce initial bias. The 10 s choice is justified by the accepted convention that the airflow cessation that lasts more than 10 s is scored as an apnea [3, 5, 31]. As such, the adaptive threshold is defined as follows:

2 $$ \begin{gathered} {\text{TH}}_{\text{adaptive}} (i) = \left\{ \begin{gathered} \theta ,\,i < 10 \hfill \\ A ,{\text{otherwise}} \hfill \\ \end{gathered} \right. \hfill \\ {\text{where}} \hfill \\ A = H\left[ {{\text{TH}}_{\text{adaptive}} (i - 1) - {\text{TI}}(i)} \right]*B(i) + \left( {1 - H\left[ {{\text{TH}}_{\text{adaptive}} (i - 1) - {\text{TI}}(i)} \right]} \right)*{\text{TH}}_{\text{adaptive}} (i - 1) \hfill \\ B(i) = (1 - \delta )*\frac{{\sum\nolimits_{k = 1}^{i - 1} {{\text{TI}}(k)} }}{i - 1} + \delta *\frac{{\sum\nolimits_{k = 1}^{i} {{\text{TI}}(k)} }}{i} \hfill \\ \end{gathered} $$

δ is the significance assigned to *i*th TI for computing the adaptive threshold *TH*_adaptive_(*i*) at the *i*th snore, and H[β] is the Heaviside step function, whose value is 0 for β < 0 (TI(*i*) > *TH*_adaptive_(*i*−1)) and 1 for β ≥ 0 (TI(*i*) ≤ *TH*_adaptive_(*i*−1)). We created two different thresholds to identify regular snores and their two snoring patterns:

LoTH_adaptive_: where the significance assigned to the *i*th TI is δ = 0.1;
HiTH_adaptive_: where the significance assigned to the *i*th TI is δ = 0.5.

We tested δ values until optimization was achieved. For all subjects, the best performance of both thresholds (LoTH_adaptive_ and HiTH_adaptive_) on identifying the two snoring patterns was achieved with the values 0.1 and 0.5, respectively.

R*egular snores* are defined as the ones for which TI(*i*) < HiTH_adaptive_(*i*). Consequently, *Non*-*regular snores* are defined as the ones for which TI(*i*) ≥ HiTH_adaptive_(*i*)*.* This work is focused on the study of regular snores.

**Fig. 2:**
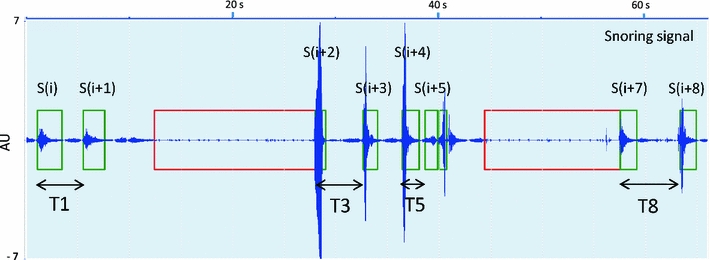
Example of an excerpt of a snoring sound signal from a subject in our database. The *small boxes* correspond to snore episodes. The *two wider boxes* correspond to two apnea episodes. T1, T3, T5 and T8 are the time intervals between the snores: *S*(*i*) and *S*(*i* + 1), *S*(*i* + 2) and *S*(*i* + 3), *S*(*i* + 4) and *S*(*i* + 5), *S*(*i* + 7) and *S*(*i* + 8), respectively

**Fig. 3:**
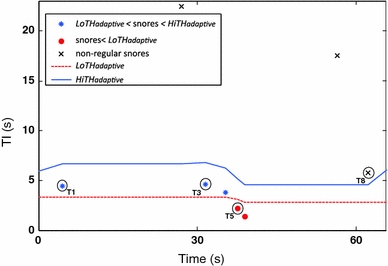
Performance of LoTH_adaptive_ and HiTH_adaptive_ on the time interval between successive snores TI(*i*) of the short segment signal shown in Fig. 2

Figure 2 shows an example of a short segment of a snoring sound signal with nine detected snores. The performance of LoTH_adaptive_ and HiTH_adaptive_ on this segment is shown in Fig. 3, where the asterisk markers under the solid line (HiTH_adaptive_) and above the dashed line (LoTH_adaptive_) correspond to snores that are selected by the combination of both thresholds. The dot markers under the dashed line (LoTH_adaptive_) are the snores selected by this lower threshold.

The time intervals between the successive snores T1, T5 and T8 correspond to: snoring pattern SP1, snoring pattern SP2 and non-regular snore, respectively. *S*(*i*) and *S*(*i* + 1) occur on two consecutive breathing cycles, so *S*(*i* + 1) corresponds to the pattern SP1. *S*(*i* + 4) and *S*(*i* + 5) occur, respectively, on the inhalation and exhalation events of the same breathing cycle and thus compose the pattern SP2. *S*(*i* + 7) is a post-apneic snore, so it is classified as a non-regular snore. Normal breathing cycles (non-snoring) occur between snores *S*(*i* + 7) and *S*(*i* + 8), hence *S*(*i* + 8) is a non-regular snore.

### Parameters and features

In this study, we analyzed a total of 74,439 snores from the whole database of 34 subjects. The method consisted in applying LoTH_adaptive_ and HiTH_adaptive_ to the all night TI(*i*) sequences to obtain the RLo*_*TI(*i*) and RMid*_*TI(*i*) sequences. These two sequences are defined as follows:

3 $$ {\text{RLo\_TI}}(i) = {\text{TI}}(i)\left| {_{{{\text{TI}}(i) < {\text{LoTH}}_{\text{adaptive}} (i)}} } \right. $$

4 $$ {\text{RMid\_TI}}(i) = {\text{TI}}(i)\left| {_{{{\text{LoTH}}_{\text{adaptive}} (i) < {\text{TI}}(i) < {\text{HiTH}}_{\text{adaptive}} (i)}} } \right. $$

From the total of 74,439 snores, after applying HiTH_adaptive_ to all 34 TI(*i*) sequences, 21,204 snores were classified as non-regular snores and 53,235 were classified as regular snores. After applying LoTH_adaptive_ to regular snores, 26,129 TI were classified as RMid*_*TI and 27,106 TI were classified as RLo*_*TI.

We calculated the mean, standard deviation and coefficient of variation for 15 min segments of the time interval sequence of regular snores. This allowed us to examine the time interval between regular snores within each 15 min segment and also during all night. Our method is similar to what is done in the field of heart rate variability (HRV), which measures time domain features of the time interval between consecutive heartbeats in small segments of a recording period [33]. In the case of HRV, the task force indicates that 5 min segments are advisable to investigate the physiological and clinical potential of HRV. The heart rate of healthy resting adults is around 60–80 beats per minute, so one can expect 300–400 beats on a 5 min recording. On the other hand, the respiratory rate in adults ranges from 12 to 20 breaths per minute [35]. Furthermore, unlike the heartbeat, snoring may not be present in each breathing cycle. As a result, a 5 min segment is very short and not effective as it will have very few snoring episodes. Bearing this in mind, we decided to apply our study to 15 min segments. This enabled us to track the changes of the snore parameters per segment and over all night.

For each *k* 15 min segment of the all night recording, we calculated three parameters both in the RLo*_*TI(*i*) and RMid*_*TI(*i*) sequences: average *μ*(*k*), standard deviation *σ*(*k*) and coefficient of variation cv(*k*) [ratio of the standard deviation *σ*(*k*) to the average *μ*(*k*)]. Thereafter, we computed the average and standard deviation of *μ*, *σ* and cv obtained for all *k* segments (Table 2). Features A*μ*, A*σ*, Acv, SD*μ*, SD*σ* and SDcv were calculated for each subject, for both RLo*_*TI(*i*) and RMid*_*TI(*i*) sequences.

**Table 2:** Features derived from the parameters

A*μ*_*X*_	Average of parameter *μ*_*X*_ over all *k* segments
A*σ*_*X*_	Average of parameter *σ*_*X*_ over all *k* segments
Acv_*X*_	Average of parameter cv_*X*_ over all *k* segments
SD*μ*_*X*_	Standard deviation of parameter *μ*_*X*_ over all *k* segments
SD*σ*_*X*_	Standard deviation of parameter *σ*_*X*_ over all *k* segments
SDcv_*X*_	Standard deviation of parameter cv_*X*_ over all *k* segments

*X* = RLo*_*TI(*i*); RMid*_*TI(*i*)

### Statistical analysis and classification

The 34 subjects were divided in two groups according to three cut-points (cp) of AHI severity: 5, 15 and 30 h^−1^. These three different levels are proposed by physicians and clinical experts as criteria for SAHS definition [21].

For each feature, the Mann–Whitney *U* test was used to assess the independence of the respective populations. Kolmogorov–Smirnov test was previously performed to confirm that the two samples had different continuous distributions [11].

We applied the Bayesian classification algorithm for supervised learning to evaluate the performance of the features on classifying the subjects according to the three abovementioned cut-points of SAHS severity [15]. To ensure the statistical validity of the classification results, we used the leave-one-patient-out cross validation process, where the training set is built by taking at each round all patients except one. All analysis in this study was executed using MATLAB^®^ (The MathWorks Inc., version 2010b).

## Results

### Screening SAHS severity using time interval between regular snores

**Fig. 4:**
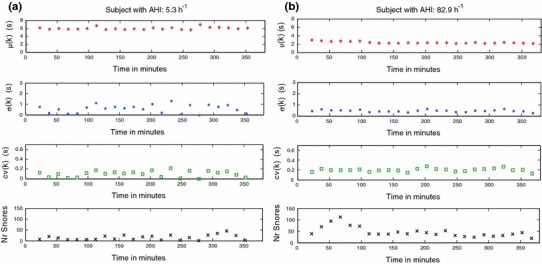
Parameters for RMid*_*TI(*i*) sequence. Parameters *μ*_RMid_TI_, *σ*_RMid_TI_ and cv_RMid_TI_ obtained for all *k* segments for two subjects with AHI **a** 5.3 h^−1^ and **b** 82.9 h^−1^

**Fig. 5:**
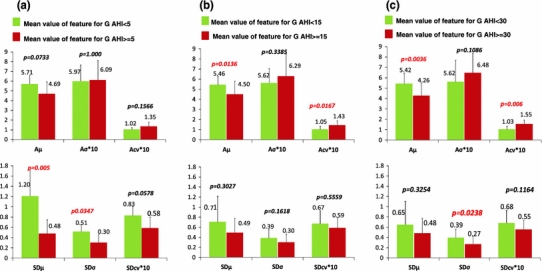
Bar graphs for features A*μ*, A*σ*, Acv, SD*μ*, SD*σ* and SDcv of RMid*_*TI for 34 subjects with three cut-points of severity: 5, 15 and 30 h^−1^. Features A*σ*, Acv and SDcv appear scaled by a factor of 10 only for the sake of a better presentation

As an illustrative example, Fig. 4 shows parameters *μ*, *σ* and cv of RMid*_*TI for each *k* 15 min segment from two subjects with opposite values of SAHS severity: (a) AHI = 5.3 h^−1^ and (b) AHI = 82.9 h^−1^. Figure 5 displays the results obtained for features A*μ*, A*σ*, Acv, SD*μ*, SD*σ* and SDcv of the RMid*_*TI sequence for all population. The bar graphs depict the mean and standard deviation values of the features for every two groups of subjects with opposite levels of AHI severity. Features A*μ*, A*σ* and Acv allow us to investigate the average of the time interval between regular snores within each *k* short segment, whereas features SD*μ*, SD*σ* and SDcv give evidence of the dispersion during all night sleep.

When examining Fig. 4a, we observe that the less severe subject (AHI of 5.3 h^−1^) presents the highest values of *μ* in each *k* segment. Figure 5 shows that less severe SAHS subjects have higher values of RMid*_*TI within each 15 min segment (A*μ*) than more severe SAHS subjects. These differences are highly statistically significant for AHI = 15 h^−1^ (*p* = 0.0136) and AHI = 30 h^−1^ (*p* = 0.0036) cut-points of severity. The variability of RMid*_*TI within each *k* segment, given by the feature Acv, is always higher for severe SAHS subjects. This feature permits distinguishing between subjects with opposite levels of severity (Fig. 5b, c, *p* = 0.0167, 0.006 for AHI cp: 15 h^−1^, 30 h^−1^; respectively).

Standard deviation of parameters *μ*, *σ* and cv enables to interpret the dispersion of time interval between consecutive snores along all night sleep. Higher variability in all three parameters is observed for the least severe subject, as compared to the same parameters in the most severe subject (Fig. 4a, b). The severe subject shows almost the same value for all three parameters in all night short *k* segments. Results obtained for the whole database confirm lower values of SD*σ* and SDcv for more severe SAHS subjects (Fig. 5), in agreement with the two individual cases shown in Fig. 4.

The results obtained for parameters and features of sequence RLo*_*TI exhibited similar behavior as the one seen for RMid*_*TI sequence. For that reason and for the sake of conciseness, results on RLo*_*TI sequence are not illustrated. For the RLo*_*TI sequence, features A*μ* (*p* = 0.0429, 0.0025; AHI cp:15 h^−1^, 30 h^−1^) and Acv (*p* = 0.0032, 0.0025; AHI cp:15 h^−1^, 30 h^−1^) enabled to distinguish between subjects with opposite levels of severity with statistical significance. For all 34 subjects and all AHI cut-points considered, SD*μ*, SD*σ* and SDcv features presented higher values for less severe subjects than for more severe subjects. This fact suggests a greater dispersion on the value of time interval between snores during all night for less severe SAHS subjects.

### Classification of subjects

Table 3 summarizes the classification results obtained with the Bayesian classifier with leave-one-patient-out cross validation process. All six features: A*μ*, A*σ*, Acv, SD*μ*, SD*σ* and SDcv were used in the classification as our purpose is to evaluate their reliability on predicting the subjects’ SAHS severity. Both RMid*_*TI and RLo*_*TI sequences obtained the best results in terms of diagnostic accuracy for 5 h^−1^ and 30 h^−1^ AHI cut-points (RMid*_*TI: accuracy = 88.2 % for AHI cp:5 h^−1^ and 73.5 % for AHI cp:30 h^−1^, RLo*_*TI: accuracy = 91.2 % for AHI cp:5 h^−1^ and 94.1 % for AHI cp:30 h^−1^). These high accuracy classification results are accompanied by a good balance of sensitivity (S) and specificity (Sp) (RMid*_*TI: S (Sp) = 90 % (75 %) for AHI cp:5 h^−1^, RLo*_*TI: S (Sp) = 94.4 % (93.8 %) for AHI cp:30 h^−1^). Even though a good accuracy value of 73.5 % was obtained for the 15 h^−1^ cp of AHI, the specificity value appears to be more compromised in the case of RLo*_*TI sequence (45.5 %).

**Table 3:** Classification results for the Bayes classifier with leave-one-patient-out cross validation

AHI cut-points	5 h^−1^			15 h^−1^			30 h^−1^		
	S	Sp	Ac	S	Sp	Ac	S	Sp	Ac
RMid*_*TI	90	75	88.2	82.6	54.6	73.5	83.3	62.5	73.5
RLo*_*TI	96.7	50	91.2	87	45.5	73.5	94.4	93.8	94.1

*S* sensitivity, *Sp* specificity, *Ac* accuracy (all in percentage)

## Discussion

The foremost ambition on the scope of SAHS is to reduce its diagnosis to the least set of biosignals as an alternative to the conventional polysomnography [6, 10]. Bearing that in mind, the latest studies have mainly focused on the analysis of ECG signals alone [19], nasal airway pressure combined with thoracic and abdominal signals [14], nocturnal pulse oximetry [37], breath sounds [20, 37] and snore sounds [9, 30]. In our study, we used uniquely the snoring sound signal collected by one microphone attached to a band around the neck. The development of simple methods such as ours, based solely on snoring sound signal analysis, should be continuously encouraged in the field of SAHS diagnosis due to the simplicity of the tracheal sound measurement and the significant information about the physiology and pathology of the airways that it contains [32].

Some research studies have already reported significant differences between post-apneic snores (snores that are produced immediately after an apnea) and all remaining snores [8, 27, 36]. Nevertheless, we consider the separation in these two groups to be insufficient. For that reason, we proposed a new methodology for classifying two distinct types of snores: *non*-*regular* and *regular* snores. Non-regular snores are the ones separated by an apnea event and/or by non-snoring breathing cycles. Regular snores are truly consecutive snores, i.e., snores that are produced in consecutive breathing cycles, without interruptions.

Cavusoglu et al. [5] and our group’s previous work [31] had tried to identify these two kinds of snores, but they did not succeed in finding a proper criterion because they considered a separation of less than 10 s to be sufficient. According to their methods, the analysis of regular snores included successive snores that are interrupted either by normal breathing cycles or by apneas that last less than 10 s. We overcame this issue by applying an adaptive threshold to the all night sequence of time interval between snores of each subject.

By applying a higher (HiTH_adaptive_) and a lower (LoTH_adaptive_) threshold we can appraise the time intervals on the two snoring patterns that comprise regular snores: the single pattern (SP1) and the double pattern (SP2). Examining the two kinds of snores classified by the application of both thresholds (RMid*_*TI snores and RLo*_*TI snores) was of major importance since it enabled to study the behavior of each feature for both kinds of snores. If we had only applied our study to regular snores altogether, we would have faced confusing and misleading outcomes that would have been much more difficult to interpret.

Results obtained for feature Acv on both sequences suggest that there is more variability in each short 15 min segment for the more severe SAHS subjects. This can be understood as intra-segment variability. This finding is in agreement with previously reported studies [31] where, in spite of not having focused on regular snores, SAHS patients showed higher snore to snore variability on intensity and frequency domain snore features (AHI cp:10 h^−1^). We must emphasize that our study has achieved similar results with no need to perform any acoustic analysis of snore episodes.

Regarding the evolution of both snoring patterns along the night, we observe that the dispersion of *μ* and *σ* (SD*μ* and SD*σ*) is much higher for less severe patients. This fact suggests that there is more variability during all sleep on this kind of patients. In addition, when examining the progress during the night of the time interval (TI) between successive regular snores, we observe less dispersion in more severe patients. This makes evidence that those patients present a steadier and shorter TI during sleep than less severe patients.

In the method proposed by Ng et al. [25], where peak frequency components via wavelet bicoherence analysis were used, the sensitivity and specificity values were reported to be 85 and 90.7 %, respectively, for differentiating between apneic and non-apneic snorers. Another fairly recently published work on multi-feature snore analysis using pitch and total airway response features [18] reported classification results of 89.3 % sensitivity with 92.3 % specificity and 90 % accuracy. One of our group’s latest published works [9] obtained performance results of 80 % sensitivity and 90.9 % specificity using a model that included intensity and frequency domain snore parameters. In contrast, using only six features derived from the analysis of time interval between snores, we achieved the best performances of 94.1 % accuracy (with 94.4 % sensitivity and 93.8 % specificity) for AHI cp of 30 h^−1^ and 88.2 % accuracy (with 90 % sensitivity and 75 % specificity) for AHI cp of 5 h^−1^. Furthermore, it should be emphasized that the complexity of the proposed method is fairly low since only the analysis of the time interval between snores is involved whereas the algorithms used in [9, 18, 25] require complex acoustic analysis of snore parameters.

Even though we used a substantial amount of snores (74,439 snores) to perform this study, an important next step will be to apply this new methodology on a wider database to confirm the results obtained in this paper. Apart from the sample size, another limitation of this study is the fact that it is only applicable to snoring subjects. Nonetheless, according to the latest publications, the non-snoring SAHS patients comprise a very small percentage of the overall spectrum of SAHS [22].

In conclusion, we designed a method that allows the identification of non-regular and regular snores. The results obtained with the features derived from the time interval between regular snores suggest that the method can be a valuable aid for the early screening and severity estimation of subjects suspected of having SAHS. In addition, it can be easily integrated in any portable and low-cost bedside monitor.
